# Influence of gender, age, and body mass index on the gut microbiota of individuals from South China

**DOI:** 10.3389/fcimb.2024.1419884

**Published:** 2024-10-31

**Authors:** Shenghui Li, Shao Fan, Yufang Ma, Chuan Xia, Qiulong Yan

**Affiliations:** ^1^ Department of Microbiology, College of Basic Medical Sciences, Dalian Medical University, Dalian, China; ^2^ Puensum Genetech Institute, Wuhan, China; ^3^ Department of Biochemistry and Molecular Biology, College of Basic Medical Sciences, Dalian Medical University, Dalian, China

**Keywords:** gut microbiota, gender, age, body mass index (BMI), metagenomics

## Abstract

**Background:**

The symbiotic gut microbiota is pivotal for human health, with its composition linked to various diseases and metabolic disorders. Despite its significance, there remains a gap in systematically evaluating how host phenotypes, such as gender, age, and body mass index (BMI), influence gut microbiota.

**Methodology/principal findings:**

We conducted an analysis of the gut microbiota of 185 Chinese adults based on whole-metagenome shotgun sequencing of fecal samples. Our investigation focused on assessing the effects of gender, age, and BMI on gut microbiota across three levels: diversity, gene/phylogenetic composition, and functional composition. Our findings suggest that these phenotypes have a minor impact on shaping the gut microbiome compared to enterotypes, they do not correlate significantly within- or between-sample diversity. We identified a substantial number of phenotype-associated genes and metagenomic linkage groups (MLGs), indicating variations in gut microflora composition. Specifically, we observed a decline in beneficial Firmicutes microbes, such as *Eubacterium*, *Roseburia*, *Faecalibacterium* and *Ruminococcus* spp., in both older individuals and those with higher BMI, while potentially harmful microbes like *Erysipelotrichaceae*, *Subdoligranulum* and *Streptococcus* spp. increased with age. Additionally, *Blautia* and *Dorea* spp. were found to increase with BMI, aligning with prior research. Surprisingly, individuals who were older or overweight exhibited a lack of Bacteroidetes, a dominant phylum in the human gut microbiota that includes opportunistic pathogens, while certain species of the well-known probiotics *Bifidobacterium* were enriched in these groups, suggesting a complex interplay of these bacteria warranting further investigation. Regarding gender, several gender-associated MLGs from *Bacteroides*, *Parabacteroides*, *Clostridium* and *Akkermansia* were enriched in females. Functional analysis revealed a multitude of phenotype-associated KEGG orthologs (KOs).

**Conclusions/significance:**

Our study underscores the influence of gender, age, and BMI on gut metagenomes, affecting both phylogenetic and functional composition. However, further investigation is needed to elucidate the precise roles of these bacteria, including both pathogens and probiotics.

## 1 Introduction

The human gut microbiota, acting as a reservoir of bacteria and genes, plays a pivotal role in host function complementarities (Qin et al., 2010; Lee et al., 2022). Additionally, it is intricately linked to various diseases and metabolic disorders such as colorectal carcinoma (Kostic et al., 2012), inflammatory bowel disease (Frank et al., 2007; Jostins et al., 2012), and type 2 diabetes (Qin et al., 2012; Yang et al., 2021). However, traditional microbiology methods have provided only a partial understanding of the human gut microbiota, often hindered by their inability to offer an unbiased representation of its complexity.

In recent years, the emergence of metagenomics has significantly advanced our understanding of the composition and function of the human gut microbiota (Gill et al., 2006; Shkoporov et al., 2019). High-throughput techniques like 16S rRNA variable region pyro-sequencing have been employed to identify microbial phylotypes, while whole-genome shotgun (WGS) sequencing of the microbial metagenome has provided insights into community composition with minimal amplification bias (Wooley et al., 2010). Recent studies have highlighted the intricate interplay among environmental factors, host phenotypes, and gut microbiota composition (Benson et al., 2010; Li et al., 2020). Notably, research by Johansen et al. revealed a more diverse virome in centenarians compared to younger and older adults, including previously undescribed viral genera (Johansen et al., 2023). Similarly, Claesson et al. observed temporal stability in fecal microbiota of the elderly, albeit with unique phylum proportions and significant variability (Claesson et al., 2011). Furthermore, Turroni et al. identified a predominance of bifidobacteria in the infant gut, along with specific co-occurrence patterns of bifidobacterial species (Turroni et al., 2012). Additionally, Yatsunenko et al. demonstrated age-associated changes in genes involved in vitamin biosynthesis and metabolism (Yatsunenko et al., 2012).

The gut microbiota of large Chinese cohorts has also been extensively studied. For examples, Zhang et al. have performed a large structural survey of fecal microbiota in 314 young adults, defining a phylo-functional core of gut microbiota, that is, the assemblage of a few bacterial genera with potentially conserved but indispensable functions for human health (Zhang et al., 2015). He et al. characterized the gut microbiota of 7009 individuals within 1 province and revealed the generalizability microbiota-based diagnostic models of metabolic disease (He et al., 2018). Winglee et al. have demonstrated that recent urbanization in China is corelated with a Westernized microbiome encoding increased virulence and antibiotic resistance genes (Winglee et al., 2017). Another research has investigated the association of Chinese gut microbiota with staple food type, ethnicity, and urbanization, providing a nationwide gut microbiota baseline of the Chinese population and knowledge on important covariates (Lu et al., 2021). Recently, several reports have emerged revealing the association between body mass index (BMI) and gut microbiota (Haro et al., 2016; Gao et al., 2018; Lv et al., 2019; Liang et al., 2023; Ren et al., 2023). Despite these insights, there remains a dearth of systematic investigation into the influence of host phenotypes, such as gender, age, and BMI, on the human gut microbiota, particularly within the Chinese adult population from South China. In this study, we re-analyzed the gut microbiota of 185 normal Chinese adults who had not taken antibiotics in the past two months from two modern cities, Shenzhen and Guangzhou, in South China. These samples were utilized to construct an updated gene catalogue, perform gene profiling, and serve as control samples in a metagenome-wide association study (MGWAS) of type 2 diabetes (Qin et al., 2012).

## 2 Results and discussion

### 2.1 Individuals, sequencing and profiling

The gut metagenomic dataset of 185 normal adults was downloaded from the NCBI Sequence Read Archive (SRA) database under project accession no. PRJNA422434, yielding a total of 345.6 gigabases (Gb) of high-quality data for analysis. On average, 74.9 ± 6.2% (mean ± SD) of reads from these samples could be accurately mapped to the gene catalogue and utilized for profiling.

The phylogenetic and functional composition of these samples was investigated by assigning genes to phylogenetic and KEGG orthologous groups (KO) via BLAST analysis, resulting in 21.3% of genes assigned to a genus and 47.1% to a KO. These assigned genes covered 58.9 ± 13.8% (mean ± SD) and 48.6 ± 4.0% of reads for genera and KOs, respectively, representing a substantial portion of the metagenomic data. Genus and KO abundance profiles in these samples were obtained by summing the relative abundances of assigned genes for each category.

### 2.2 Characteristics of the gut microbiota

**Figure 1:**
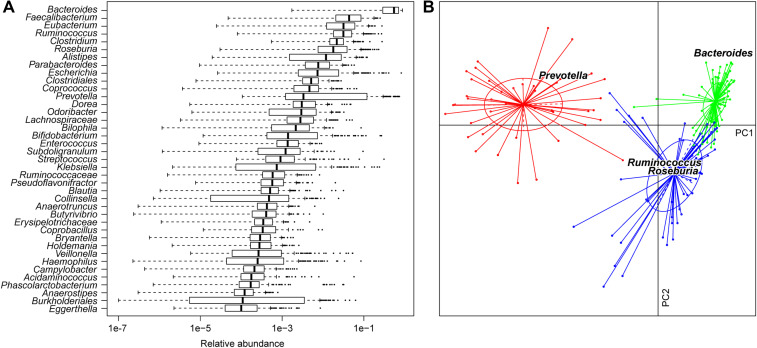
Characteristics of the gut microbiota of South Chinese adult individuals. **(A)** Phylogenetic composition of the 185 samples illustrated by the top 40 abundance genera. Boxes denote the interquartile range (IQR) between the first and third quartiles (25th and 75th percentiles, respectively) and the line inside denotes the median. Whiskers denote the lowest and highest values within 1.5 times IQR from the first and third quartiles, respectively. Circles denote outliers beyond the whiskers. **(B)** Enterotypes of Chinese samples. Based on principal component analysis, these samples were plotted on the first two principal components of the genus profile. Lines connect individuals determined to have the same enterotype, and colored circles cover the individuals near the center of gravity for each cluster (<1.5σ). The top four genera as the main contributors to these clusters were determined and plotted by their loadings in these two components.

Zhang et al. analyzed gut microbiome from fecal samples of healthy adults in three cities across China, providing a basis for understanding gut microbiome composition in certain Chinese populations (Zhang et al., 2019). Their study included samples from 131 individuals across three geographical regions (Beijing in the north, Jinan in the east, and Zigong in the southwest). However, only 11 samples underwent whole-metagenome shotgun sequencing (WMS), with the majority subjected to 16S rDNA sequencing. In contrast, our study was based on comprehensive WMS for all 185 samples, focusing on a single population from South China, and includes a larger sample size. The phylogenetic composition of these samples is depicted through genus profiles (**Figure 1A**). Major taxa included Bacteroidetes (predominantly *Bacteroides*, *Prevotella*, and *Alistipes*), Firmicutes (*Faecalibacterium*, *Eubacterium*, and *Ruminococcus*), Proteobacteria (*Escherichia* and *Klebsiella*), and Actinobacteria (*Bifidobacterium*), reflecting microbiota compositions observed in European and American populations (Zoetendal et al., 2008; Human Microbiome Project, 2012; Zhernakova et al., 2016). Many studies have indicated that human gut microbiome composition is primarily influenced by enterotypes, a subclassification unrelated to nationality, gender, age, or health conditions (Arumugam et al., 2011; Lai et al., 2023). In our dataset, enterotypes were classified based on genus composition and corroborated by KO profiles (**Figure 1B**; **Supplementary Figure S1**). Comparative analysis with European enterotypes showed that, among Chinese individuals from South China, *Roseburia* had a higher contribution than *Ruminococcus* in enterotype 3.

We found no association between enterotypes and the phenotypes of gender, age, and BMI, which is consistent with prior research (**Supplementary Table S1**) (Arumugam et al., 2011; Wu et al., 2011; Lai et al., 2023). The subsequent permutational multivariate analysis of variance (PERMANOVA) revealed that enterotypes explained the most variation in both gene and KO profiles. However, gender, age, and BMI also accounted for minor variations in these profiles (**Table 1**). Notably, significant P-values for age (*P* = 0.015, 1,000 permutations) and BMI (*P* = 0.074) were observed in gene profiles. Additionally, age (*P* = 0.016) exhibited a significant association in the KO profile, underscoring the notable impact of these phenotypes on gut microbiota.

**Table 1:** PERMANOVA analysis for the gene and KO profiles.

Phenotypes	No. of subjects	R^2^ (*P*) for gene profiles	R^2^ (*P*) for KO profiles
Gender	2	0.57% (0.153)	0.41% (0.341)
Age	51	0.80% (0.015)	1.09% (0.016)
BMI	156	0.64% (0.074)	0.43% (0.313)
Enterotypes	3	12.9% (<1x10^-4^)	29.1% (<1x10^-4^)

R^2^ and P-values were adjusted for multiple comparisons.

### 2.3 Gut microbiome biodiversity is steady across different phenotypes in adults

Biodiversity serves as a metric for assessing gut microbiome richness and evenness, often correlating with host phenotypes. Previous studies have associated biodiversity with obesity and age, as well as geographical location (Yatsunenko et al., 2012), while our dataset yielded consistent results. Two estimators, gene count (unweighted) and Shannon index (weighted), revealed a high level of within-sample diversity across samples. A high gene count (range from 105.0K to 795.4K) and Shannon index (range from 9.65 to 12.41) were observed in these samples, moreover, these two estimators showed a positive correlation because of calculation method (**Supplementary Figure S2**). Notably, this diversity exhibited no significant correlation with gender, age, or BMI (**Table 2**), except for differences among enterotypes. Particularly, enterotype 3 demonstrated higher diversity in comparison to the other two enterotypes, indicating a more even distribution of species within this enterotype.

**Table 2:** Within-sample diversity of the gut metagenomes.

	Groups (No. of samples)	Gene count (unweighted)	Shannon index (weighted)	Shannon index (KO profiles)
**Gender**	female (90)	452,583 ± 146,686	11.46 ± 0.54	4.02 ± 0.32
	male (95)	444,818 ± 128,558	11.34 ± 0.56	4.06 ± 0.37
	*P* (Student’s t-test)	0.7029	0.1177	0.3652
**Age**	<45 (younger, 98)	464,085 ± 135,460	11.46 ± 0.56	3.96 ± 0.38
	>=45 (older, 87)	431,247 ± 138,152	11.33 ± 0.53	4.13 ± 0.30
	*P* (Student’s t-test)	0.1041	0.1207	0.0095
**BMI**	<20 (lean, 48)	474,818 ± 141,246	11.47 ± 0.58	3.92 ± 0.37
	20~25 (middle-weight, 73)	440,079 ± 141,295	11.35 ± 0.57	4.12 ± 0.31
	>=25 (over-weight, 64)	438,643 ± 129,185	11.39 ± 0.51	4.02 ± 0.34
	*P* (Kruskal-Wallis test)	0.3869	0.3462	0.0126
**Enterotypes**	1 (*Bacteroides*, 83)	416,035 ± 125,269	11.45 ± 0.40	3.90 ± 0.29
	2 (*Prevotella*, 43)	450,381 ± 129,584	10.92 ± 0.65	4.24 ± 0.34
	3 (*Roseburia/Ruminococcus*, 59)	493,100 ± 148,223	11.68 ± 0.43	4.04 ± 0.33
	*P* (Kruskal-Wallis test)	0.0081	5.05x10^-9^	1.06x10^-8^

Furthermore, the Shannon index was utilized to gauge functional-level diversity based on KO profiles, revealing increased diversity among older individuals (*P* = 0.0095, Student’s t-test) and/or those with intermediate BMI (*P* = 0.013, Kruskal-Wallis test). Assessment of between-sample diversity via beta diversity (unweighted) and Hellinger distance (weighted) unveiled no significant correlation with gender, age, or BMI (**Supplementary Figure S3**). Noteworthy is the considerable divergence among enterotypes. Overall, these findings highlight the relatively stable biodiversity of gut microbiota across gender, age, and BMI in adult individuals from South China.

### 2.4 Genes and phylogenetic variation of gut microbiome

As depicted in **Supplementary Figure S4**, the distribution of p-values for genes across each phenotype revealed a substantial proportion of genes conforming to the null hypothesis, indicative of detectable gene variations within our dataset. We identified 178,024 genes associated with age (*P* ≤ 0.05, corresponding to 44.6% FDR), 163,788 associated with BMI (*P* ≤ 0.05, 45.8% FDR), and 35,850 associated with gender (*P* ≤ 0.01, 48.5% FDR) (**Supplementary Tables S2**, **S3**). To manage the extensive data and facilitate taxonomic categorization, genes were further clustered into metagenomic linkage groups (MLGs).

#### 2.4.1 Age-associated MLGs

Regarding age, we identified 237 MLGs (comprising ≥50 genes), among which 137 exhibited a decrease with aging, while 100 showed an increase. Of these MLGs, 131 were classified at phylogenetic levels ranging from order to species (**Table 3**). Notably, MLGs affiliated with the genera Alistipes, Bacteroides, and Parabacteroides, all within the phylum Bacteroidetes, demonstrated a tendency to decrease with age, with 25 out of 33 MLGs (containing 10,181 of 12,133 genes) exhibiting this pattern. While Bacteroidetes, predominant in the human gut microbiota, are commonly regarded as opportunistic pathogens, their role in aging remains enigmatic and warrants further elucidation (Vaiserman et al., 2020; Pan et al., 2023). Additionally, a prominent member of this phylum, *Bacteroides thetaiotaomicron*, was observed to increase with age, potentially serving to inhibit the activation of pro-inflammatory transcription factors in elderly individuals (Xu et al., 2003; Kelly et al., 2004).

**Table 3:** Statistics of phylogenetic levels of age-associated MLGs.

Phyla	Families/genera	Decrease with aging		Increase with aging			Species of MLGs
		# MLGs	# genes	# MLGs	# genes		
Bacteroidetes	*Rikenellaceae/Alistipes*	4	343			↓	*A. putredinis, A. shahii, Alistipes* sp. HGB5*
	*Bacteroidaceae/Bacteroides*	19	8,691	7	1,888	↓	*B. finegoldii, B. intestinalis, B. ovatus, B. stercoris, B. vulgatus, Bacteroides* sp. *1_1_6, Bacteroides* sp. *D20 decrease with aging, B. coprophilus, B. plebeius, B. thetaiotaomicron increase with aging*
	*Porphyromonadaceae/Parabacteroides*	2	1,147	1	64	↓	*P. merdae*
Firmicutes	*Acidaminococcaceae/Acidaminococcus*			2	337	↑	*A. fermentans*
	*Clostridiaceae/Clostridium*			15	7,242	↑	*C. bolteae, C. hathewayi, C. leptum, C. perfringens, C. scindens, C. symbiosum, Clostridium* sp. *HGF2*
	*Erysipelotrichaceae*	1	75	4	2329	↑	*Coprobacillus* sp. *29_1, Coprobacillus* sp. *D7, Clostridium ramosum*
	*Lactobacillaceae/Lactobacillus*			2	918	↑	L. mucosae*
	*Ruminococcaceae/Subdoligranulum*			2	1,987	↑	*Subdoligranulum* sp. 4_3_54A2FAA*
	*Streptococcaceae/Streptococcus*	1	116	5	2,447	↑	*S.parasanguinis, S. salivarius, Streptococcus* sp. *73H25AP, S. vestibularis increase with aging, S. bovis derease with aging*
	*Veillonellaceae/Veillonella*			1	984	↑	*V. parvula*
	*Eubacteriaceae/Eubacterium*	3	1,237			↓	*E. eligens, E. hallii, E. siraeum*
	*Lachnospiraceae/Roseburia*	1	446			↓	*R. inulinivorans*
	*Ruminococcaceae/Faecalibacterium*	10	5,616			↓	*F. prausnitzii*
	*Ruminococcaceae/Ruminococcus*	7	6,976	3	291	↓	*R. obeum, Ruminococcus* sp. *5_1_39BFAA, Ruminococcus* sp. *SR1/5 decrease with aging, R. bromii, R. gnavus increase with aging*
Fusobacteria	*Fusobacterium mortiferum*			1	184	↑	
	*Fusobacterium ulcerans*			3	484	↑	
	*Fusobacterium varium*	1	1,012			↓	
Proteobacteria	*Burkholderiales*	4	1,504			↓	*Burkholderiales bacterium 1_1_47*
	*Enterobacteriaceae/Escherichia*			2	143	↑	*E. coli*
	*Enterobacteriaceae/Klebsiella*			3	441	↑	*K. pneumoniae*
Actinobacteria	*Bifidobacteriaceae/Bifidobacterium*			3	1,828	↑	*B. dentium, B. longum, B. pseudocatenulatum*

MLGs were assigned into phylogenetic levels at the nucleotide level or, when marked with * at the protein level.

Significant variability with aging was also observed among MLGs affiliated with the phylum Firmicutes. At the genus level, MLGs from *Clostridium, Erysipelotrichaceae*, *Lactobacillus, Subdoligranulum*, *Streptococcus*, and *Veillonella* spp. notably increased with age, whereas MLGs from *Eubacterium*, *Roseburia*, *Faecalibacterium*, and *Ruminococcus* spp. decreased (**Table 3**). Notably, the age-decreased bacteria predominantly belong to beneficial species such as *Eubacterium hallii*, *Roseburia inulinivorans* (both recognized as butyrate producers (Duncan et al., 2004; Duncan et al., 2006; Engels et al., 2016)), and *Ruminococcus obeum* (Hayashi et al., 2003). Particularly striking was the decline observed in the genus *Faecalibacterium* (predominantly represented by *F. prausnitzii*), which exhibited a significant decrease across different age stages (r=-0.30 with age, *P* = 2.53 x 10^-4^, Spearman’s rho correlation coefficient test; **Supplementary Figure S5**), consistent with findings from other investigations (Biagi et al., 2010; Kim and Jazwinski, 2018; Donati Zeppa et al., 2022). *Faecalibacterium* exerts beneficial effects such as butyrate production and modulation of gut inflammation processes (Guo et al., 2023; Martin et al., 2023).

Moreover, several MLGs from pathogen-like genera such as *Escherichia* and *Klebsiella* (both within the phylum Proteobacteria) demonstrated an increase with aging. However, MLGs from *Bifidobacterium* (specifically *B. dentium* and *B. longum*) - well-known probiotic genera - also exhibited an upward trend with age (refer to **Table 3**). While *B. dentium* has been documented as an opportunistic oral pathogen (Ventura et al., 2009), *B. longum* displayed a significant increase between youth and older individuals in our study (*P* = 0.031), despite a recorded decline in adults compared to children/infants (Turroni et al., 2012). Additionally, *Fusobacterium*, commonly associated with various human diseases (Stavreas et al., 2005; Huggan and Murdoch, 2008; Stokowa-Soltys et al., 2021), displayed a positive correlation with age, with *F. mortiferum* and *F. ulcerans* showing an increase, while *F. varium* exhibited a decrease (**Table 3**).

#### 2.4.2 BMI-associated MLGs

We identified 215 MLGs (comprising ≥50 genes), with 172 showing decreased abundance and 43 exhibiting increased abundance in association with BMI. Among these, 79 MLGs were categorized into phylogenetic levels. Notably, within the phylum Bacteroidetes, MLGs originating from *Alistipes*, *Bacteroides*, and *Odoribacter* spp. demonstrated significant decreases in abundance with increasing BMI, whereas MLGs from *Prevotella* exhibited an increase (**Table 4**). Specifically, 16 out of 18 MLGs derived from *Bacteroides* spp., encompassing 8,979 out of 8,936 genes, showed decreased abundance with increasing BMI. Conversely, the remaining 2 MLGs, including *B. vulgatus*, an opportunistic pathogen associated with peritoneal diseases, showed increased abundance, potentially exerting detrimental effects on individuals with higher body weight (Bamba et al., 1995; Shiba et al., 2003; Mills et al., 2022; Pan et al., 2023).

**Table 4:** Statistics of phylogenetic levels of BMI-associated MLGs.

Phyla	Families/genera	Decrease with BMI		Increase with BMI			Species of MLGs
		# MLGs	# genes	# MLGs	# genes		
Bacteroidetes	*Rikenellaceae/Alistipes*	4	2,358			↓	*A. putredinis, A. shahii*
	*Bacteroidaceae/Bacteroides*	16	8,979	2	857	↓	*B. cellulosilyticus, B. coprocola, B. eggerthii, B. intestinalis, B. ovatus, B. uniformis, B. xylanisolvens derease with BMI, B. vulgatus increase wich BMI*
	*Porphyromonadaceae/Odoribacter*	1	1,749			↓	*O. splanchnicus*
	*Prevotellaceae/Prevotella*			2	290	↑	*P. copri*
Firmicutes	*Lachnospiraceae/Blautia*			1	429	↑	*B. hansenii*
	*Lachnospiraceae/Dorea*			3	684	↑	*D. formicigenerans, D. longicatena*
	*Eubacteriaceae/Eubacterium*	1	402			↓	*E. eligens*
	*Lachnospiraceae/Roseburia*	3	396			↓	*R. intestinalis, R. inulinivorans*
	*Ruminococcaceae/Faecalibacterium*	8	4,260			↓	*F. prausnitzii*
	*Ruminococcaceae/Ruminococcus gnavus*			2	2,885	↑	
	*Ruminococcaceae/other Ruminococcus*	5	3,478			↓	*R. bromii, R. lactaris, R. obeum, Ruminococcus* sp. *SR1/5*
Actinobacteria	*Bifidobacteriaceae/Bifidobacterium*			2	2,642	↑	*B. bifidum, B. pseudocatenulatum*

Within the phylum Firmicutes, MLGs from *Blautia*, *Dorea*, and *Ruminococcus gnavus* demonstrated increased abundance with increasing BMI, whereas MLGs from *Eubacterium*, *Roseburia*, *Faecalibacterium*, and *Ruminococcus* spp. (excluding *R. gnavus*) showed decreased abundance (**Table 4**). Noteworthy is the observation that MLGs from *Ruminococcus gnavus*, a mucolytic bacterium associated with the colon, exhibited increased abundance with both aging and increasing BMI, suggesting a potential role in these contexts. Similarly, akin to aging, bacteria exhibiting decreased abundance with increasing BMI included several known beneficial species, such as *Roseburia intestinalis*, *Roseburia inulinivorans*, *Faecalibacterium prausnitzii*, and various *Ruminococcus* species.

Furthermore, two MLGs from the genus *Bifidobacterium*, namely *B. bifidum* and *B. pseudocatenulatum*, displayed increased abundance with increasing BMI (**Table 4**). Numerous studies have highlighted the beneficial effects of these *Bifidobacterium* species (Saavedra et al., 1994; Turroni et al., 2019; Lee et al., 2021). We acknowledge that some of the studies have come to different conclusions about the relationship between the abundance of *Bifidobacterium* and age or BMI, which may due to sample size, regional differences, or individual dietary habits (Zimmermann and Curtis, 2020; Hassan et al., 2022; Yoshida et al., 2022; Escouto et al., 2023). Nevertheless, further investigation is warranted to elucidate their specific roles.

#### 2.4.3 Gender-associated MLGs

We subsequently identified 43 MLGs (comprising ≥50 genes), with 29 enriched in females and 14 enriched in males, from gender-associated genes. Among these, 29 MLGs were assigned into phylogenetic levels. Interestingly, MLGs from *Bacteroides*, *Parabacteroides*, *Clostridium*, and *Akkermansia* spp. were all enriched in females (**Table 5**). This finding contrasts with previous studies (Mueller et al., 2006; Li et al., 2008) that reported higher levels of Bacteroides and Clostridia in males compared to females. It is plausible that gender influences susceptibility to the effects of microbiota (Davey et al., 2012).

**Table 5:** Statistics of phylogenetic levels of gender-associated MLGs.

Phyla	Families/genera	Enriched in female		Enriched in male		Species of MLGs
		# MLGs	# genes	# MLGs	# genes	
Bacteroidetes	*Bacteroidaceae/Bacteroides*	10	6,495			*B. cellulosilyticus, B. ovatus, B. stercoris, B. thetaiotaomicron, B. uniformis, Bacteroides* sp. *3_1_23, Bacteroides* sp. *3_1_33FAA*
	*Porphyromonadaceae/Parabacteroides*	1	1,161			*P. merdae*
Firmicutes	*Clostridiaceae/Clostridium*	3	788			*C. bolteae, C. symbiosum, Clostridium* sp. *HGF2*
Verrucomicrobia	*Verrucomicrobiaceae/Akkermansia*	2	2,147			A. muciniphila*

### 2.5 Functional variation of gut microbiome

We investigated the functional variation of gut microbiota under the influence of various phenotypes through association analysis based on KO (KEGG ortholog) relative abundance profiles. Using analogous methods as those employed for gene profiles, we identified 631 age-associated KOs (*P* ≤ 0.05, corresponding to 28.9% FDR), 251 BMI-associated KOs (*P* ≤ 0.05, 100% FDR), and 439 gender-associated KOs (*P* ≤ 0.05, 52.4% FDR) (**Supplementary Table S** **4**).

**Figure 2:**
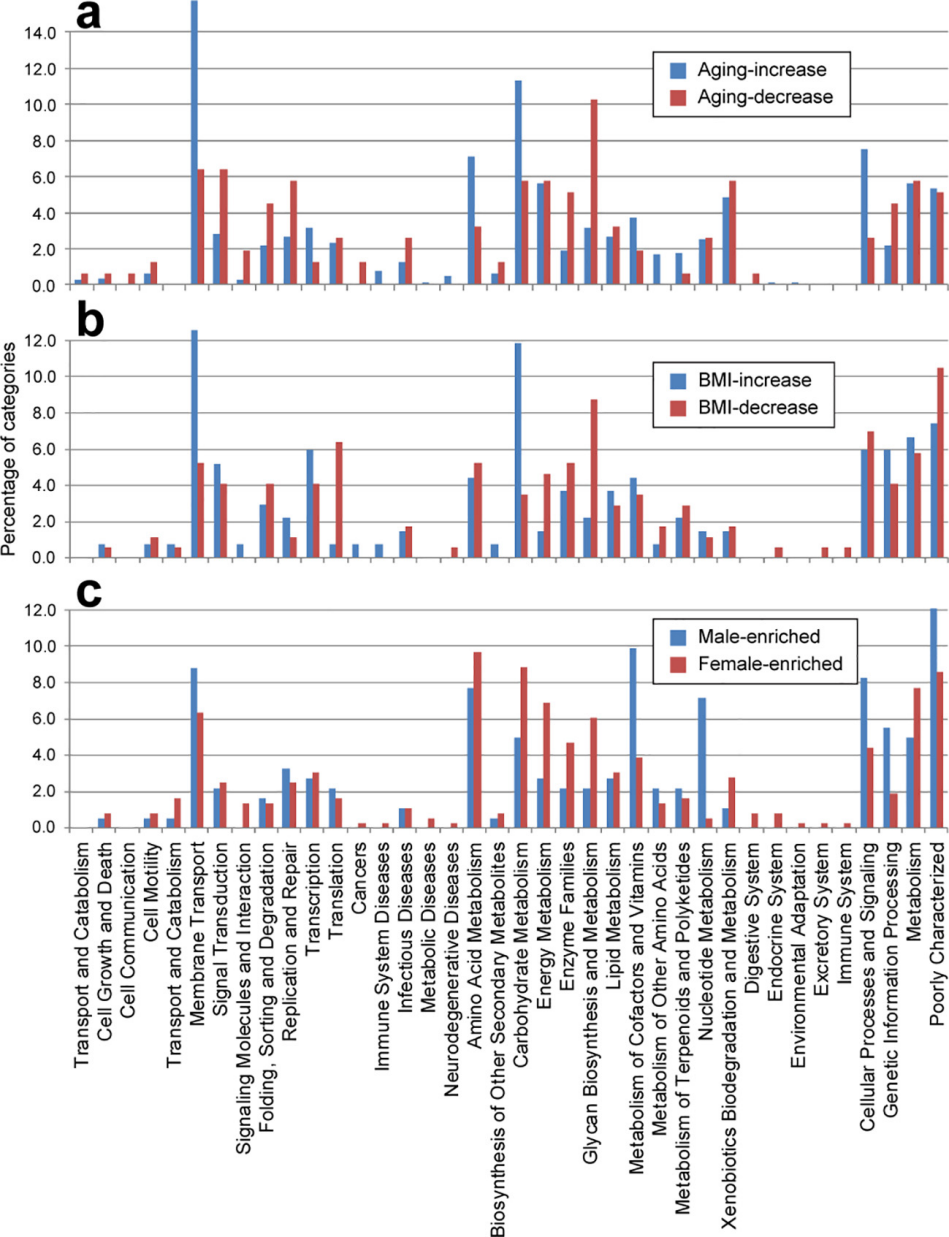
Distribution of functional categories (level B) for phenotype-associated KEGG orthologs. Bar plot showing the comparisons of age-associated **(A)**, BMI-associated **(B)**, and gender-associated **(C)** KOs.

In terms of aging, KOs that increased with age were enriched in categories related to “membrane transport”, “amino acid metabolism”, and “carbohydrate metabolism”, while those that decreased were enriched in “signal transduction”, “DNA replication and repair”, “enzyme families”, and “glycan biosynthesis and metabolism” (**Figure 2A**). These conclusions are consistent with the reported findings. For instance, metabolism of aromatic amino acids are shown to positively associated with aging (Rampelli et al., 2013; Wu et al., 2019); Older adults are proven to have a reduced number of gene families involved in genetic transcription, repair and defense mechanisms compared to younger people (Odamaki et al., 2018); glycosylation has been demonstrated to be associated with aging (Kobata, 2003; Dall'Olio, 2018; Cindric et al., 2021). However, Older adults were reported to have reduced pathway related to carbohydrate metabolism (Rampelli et al., 2013; Odamaki et al., 2018), which contradict with our data. Similarly, for BMI, KOs that increased with BMI were enriched in “membrane transport” and “carbohydrate metabolism”, whereas those that decreased were enriched in “translation”, “energy metabolism”, and “glycan biosynthesis and metabolism” (**Figure 2B**). The observation of elevated levels of membrane transport in the gut microbiota across several diseases, including obesity, inflammatory bowel disease (Greenblum et al., 2012), and type 2 diabetes (Zhai et al., 2021), aligns with our findings, suggesting potential adverse effects associated with aging and higher BMI. Additionally, categories such as carbohydrate and amino acid metabolism were upregulated in older and overweight individuals, indicative of an enhanced capacity for energy harvest in these populations (Turnbaugh et al., 2006; Turnbaugh et al., 2009). Conversely, the category of glycan biosynthesis and metabolism, particularly glycosyltransferases and lipopolysaccharide biosynthesis proteins, exhibited significant depletion in older and overweight individuals, suggesting favorable effects associated with these functions.

Regarding gender, females showed enrichment in “carbohydrate metabolism”, “energy metabolism”, “enzyme families”, and “glycan biosynthesis and metabolism”, whereas males exhibited enrichment in “nucleotide metabolism” and “metabolism of cofactors and vitamins” (**Figure 2C**).

Research on the gut microbiota of Chinses people have continuously emerged (Zhang et al., 2015; Haro et al., 2016; Winglee et al., 2017; Gao et al., 2018; He et al., 2018; Lv et al., 2019; Zhang et al., 2019; Lu et al., 2021; Liang et al., 2023; Ren et al., 2023). In comparison to the published studies, our findings are consistent with the general consensus that age, BMI, and gender do influence the gut microbiome, although the effect size may be smaller compared to other factors such as diet and enterotypes. For instance, previous studies have shown significant shifts in gut microbiota composition with age and BMI, aligning with our observation of changes in specific microbial taxa and functional genes. Importantly, our study adds to the literature by focusing on a unique population from South China, thereby highlighting regional differences that may contribute to these variations. Although our study provides valuable insights into the influence of gender, age, and BMI on the gut microbiomes of individuals from South China, we acknowledge that the sample size of 185 adults may not be large enough to fully represent the entire population. Future studies with larger and more diverse cohorts are needed to validate and extend our findings.

## 3 Materials and methods

### 3.1 Data sources, sequencing and profiling

To characterize the gut microbiota of typical adult individuals from South China, we re-analyzed the gut metagenomic dataset of fecal samples from 185 subjects (90 females and 95 males), aged 14 to 74 years, with BMI ranging from 15.6 to 32.6 kg/m^2^. The raw metagenomic sequencing read data of all samples was downloaded from the NCBI SRA database with accession no. PRJNA422434. These individuals had not taken antibiotics in the past two months, and their fecal samples were frozen immediately and underwent DNA extraction with standard methods (Godon et al., 1997). Adapter contamination and low-quality reads were discarded from the raw reads, and the remaining reads were filtered to eliminate human host DNA based on the human genome reference (hg18).

A gene catalogue, amalgamated from European and Chinese cohorts, comprising nearly 4.3 million genes, was utilized for analysis (Qin et al., 2010; Qin et al., 2012). The gene abundance in the 185 samples was quantified using SOAP2, a rapid short read alignment tool (Li et al., 2009), with a similarity threshold set at 90%. The quantitative relative abundance of each gene was calculated by normalizing the number of reads mapped to each gene by their respective lengths. To mitigate the influence of varying sequence amounts among samples, gene relative abundances were normalized within each sample.

To explore the impact of gender, age, and BMI on gut microbiomes, we performed PERMANOVA analysis based on gene and KEGG ortholog (KO) profiles. We assessed the association between enterotypes and these phenotypes and evaluated the extent to which enterotypes, gender, age, and BMI explained variation in these profiles.

### 3.2 Biodiversity and phylogenetic variation assessment

Biodiversity serves as a metric for assessing gut microbiome richness and evenness, often correlating with host phenotypes. To evaluate biodiversity, we used two estimators: gene count (unweighted) and Shannon index (weighted). The gene count provides an unweighted measure of the total number of genes present, while the Shannon index accounts for both richness and evenness of species distribution. For functional-level diversity, we used the Shannon index based on KEGG ortholog (KO) profiles. Beta diversity was assessed using unweighted and Hellinger distances to measure between-sample diversity.

To comprehensively explore the gene and phylogenetic variations within the gut microbiome under the influence of gender, age, and BMI, we conducted association analyses to identify differential genes associated with each phenotype based on gene abundance profiles. Initially, to mitigate potential biases stemming from other phenotypes, we employed stratified random sampling among the 185 samples (or population stratification akin to GWAS), yielding a subset of samples for each phenotype (**Supplementary Table S2**).

Genes occurring in fewer than six samples were excluded. The remaining genes underwent analysis of abundance profiles using Student’s t-test for gender and Spearman’s rho correlation coefficient test for age and BMI. Integrating p-values with the false discovery rate (FDR), we identified genes associated with each phenotype. Genes were further clustered into metagenomic linkage groups (MLGs) using previously described methods (Qin et al., 2012; Wang et al., 2022) to manage the extensive data and facilitate taxonomic categorization.

### 3.3 MLG identification and taxonomy assignment

#### 3.3.1 Clustering methods for identifying MLG

In this study, a concept of metagenomic linkage group (MLG), which could facilitate the taxonomic description of metagenomic data from whole-genome shotgun sequencing were devised. To identify MLG from the set of T2D-associated gene markers, we developed an in-house software that comprises three steps as indicated below:

Step 1: The original set of T2D-associated gene markers was taken as initial sub-clusters of genes. It should be noted that in the establishment of the gene profile we had constructed gene linkage groups to reduce the dimensionality of the statistical analysis. Accordingly, all genes from a gene linkage group were considered as one sub-cluster.

Step 2: We applied the Chameleon algorithm (Yao et al., 2023) to combine the sub-clusters exhibiting a minimal similarity of 0.4 using dynamic modeling technology and basing selection on both interconnectivity and closeness. The similarity here is defined by the product of interconnectivity and closeness (we used this definition in the whole analysis of MLG identification). We term these clusters semi-clusters.

Step 3: To further merge the semi-clusters established in step 2. In this step, we first updated the similarity between any two semi-clusters, and then performed a taxonomic assignment for each semi-cluster (see the method below). Finally, two or more semi-clusters would be merged into a MLG if they satisfied both of the following two requirements: a) the similarity values between the semi-clusters were > 0.2; b) all these semi-clusters were assigned from the same taxonomy lineage.

#### 3.3.2 Taxonomic assignment for MLGs

All genes from one MLG were aligned to the reference microbial genomes (IMG database) at the nucleotide level (by BLASTN) and the NCBI-NR database at the protein level (by BLASTP). The alignment hits were filtered by both e-value (< 1×10^-10^ at the nucleotide level and < 1×10^-5^ at the protein level) and alignment coverage (>70% of a query sequence). From the alignments with the reference microbial genomes, we obtained a list of well-mapped bacterial genomes for each MLG and ordered these bacterial genomes according to the proportion of genes that could be mapped onto the bacterial genome, as well as the average identity of the alignments. The taxonomic assignment of a MLG was determined by following principles: 1) if more than 90% of genes in this MLG can be mapped onto a reference genome with a threshold of 95% identity at the nucleotide level, this particular MLG was considered to originate from this known bacterial species; 2) if more than 80% of genes in this MLG can be mapped onto a reference genome with a threshold of 85% identity at the both nucleotide and protein levels, this MLG were considered to originate from the same genus of the matched bacterial species; 3) if the 16S rDNA sequences can be identified from the assembly result of a MLG, the phylogenetic analysis by RDP-classifier (Wang et al., 2007) (bootstrap value > 0.80) were preformed, and then the taxonomic assignment for the MLG was defined if the phylotype from 16S sequences was consistent with that from genes.

#### 3.3.3 Statistical analyses

Statistical analyses were implemented using the R software environment. Permutational multivariate analysis of variance (PERMANOVA) was conducted using the adonis function from the vegan package to determine the effect of host properties on the gut microbiome. The Student’s t-test and Kruskal-Wallis test were employed to analyze differences in Shannon index among different groups. Statistical significance was determined at a *p*-value threshold of < 0.05, and the *q*-value was computed to adjust for the false discovery rate (FDR) for multiple comparisons.

## Data Availability

Publicly available datasets were analyzed in this study. This data can be found here: NCBI SRA database with accession no. PRJNA422434.
