# Supplementary material for: Influence of gender, age, and body mass index on the gut microbiota of individuals from South China
Source: Front Cell Infect Microbiol. 2024 Oct 31;14:1419884. doi: 10.3389/fcimb.2024.1419884 (PMC11560914; doi:10.3389/fcimb.2024.1419884)
Supplement: Supplementary file 1 [file Table1.docx]

Supplementary Material

Influence of Gender, Age, and Body Mass Index on the Gut Microbiota of Individuals from South China

Shenghui Li^1,2#^, Shao Fan^1,3#^, Yufang Ma^1,3^, Chuan Xia^1*^, Qiulong Yan^1*^

^1^Department of Microbiology, College of Basic Medical Sciences, Dalian Medical University, Dalian, China

^2^Puensum Genetech Institute, Wuhan, China

^3^Department of Biochemistry and Molecular Biology, College of Basic Medical Sciences, Dalian Medical University, Dalian, China

^#^These authors contributed equally to this work

***Correspondence:**Chuan Xia: xiachuan@dmu.edu.cn; Qiulong Yan: qiulongy1988@163.com

# Supplementary Figures


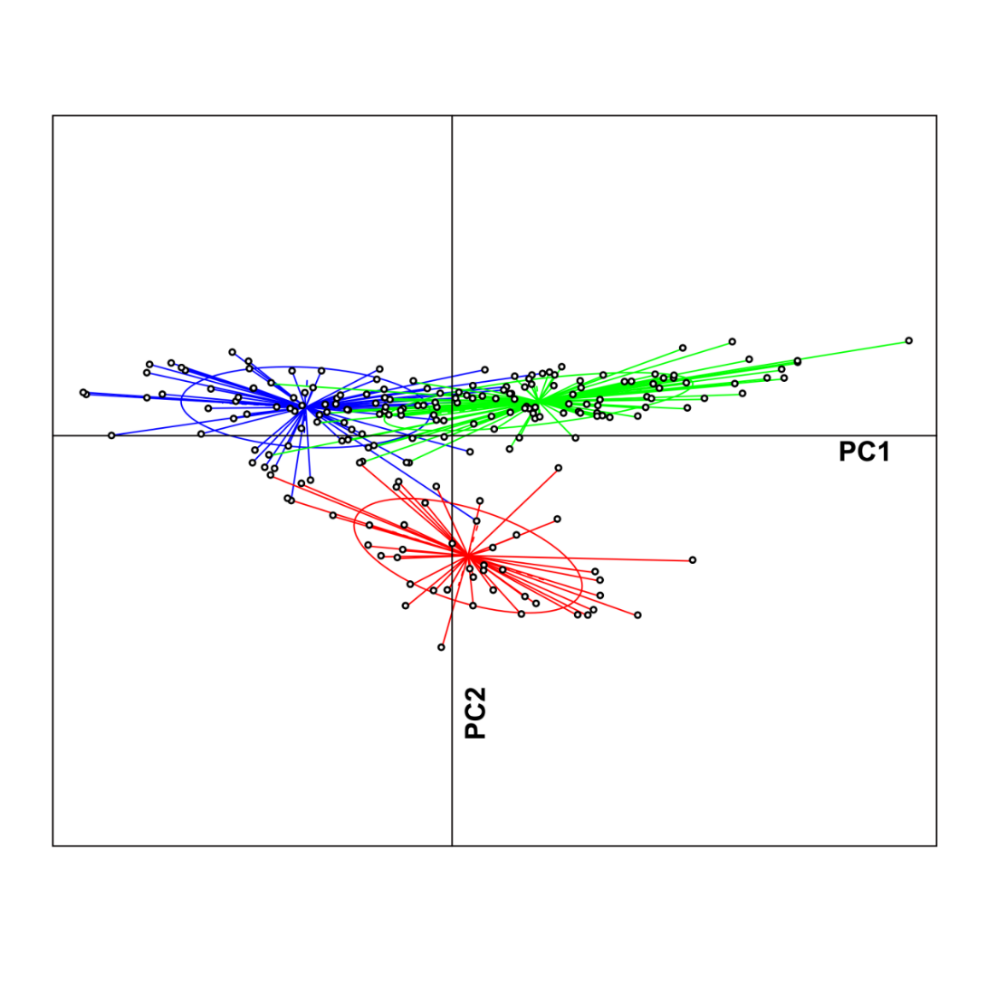


**Supplementary Figure 1.** Principal Component Analysis (PCA) of KO profiles of 185 Chinese samples. Three enterotypes were labeled and grouped with colored circles (red: enterotype 1; green: enterotype 2; blue: enterotype 3).


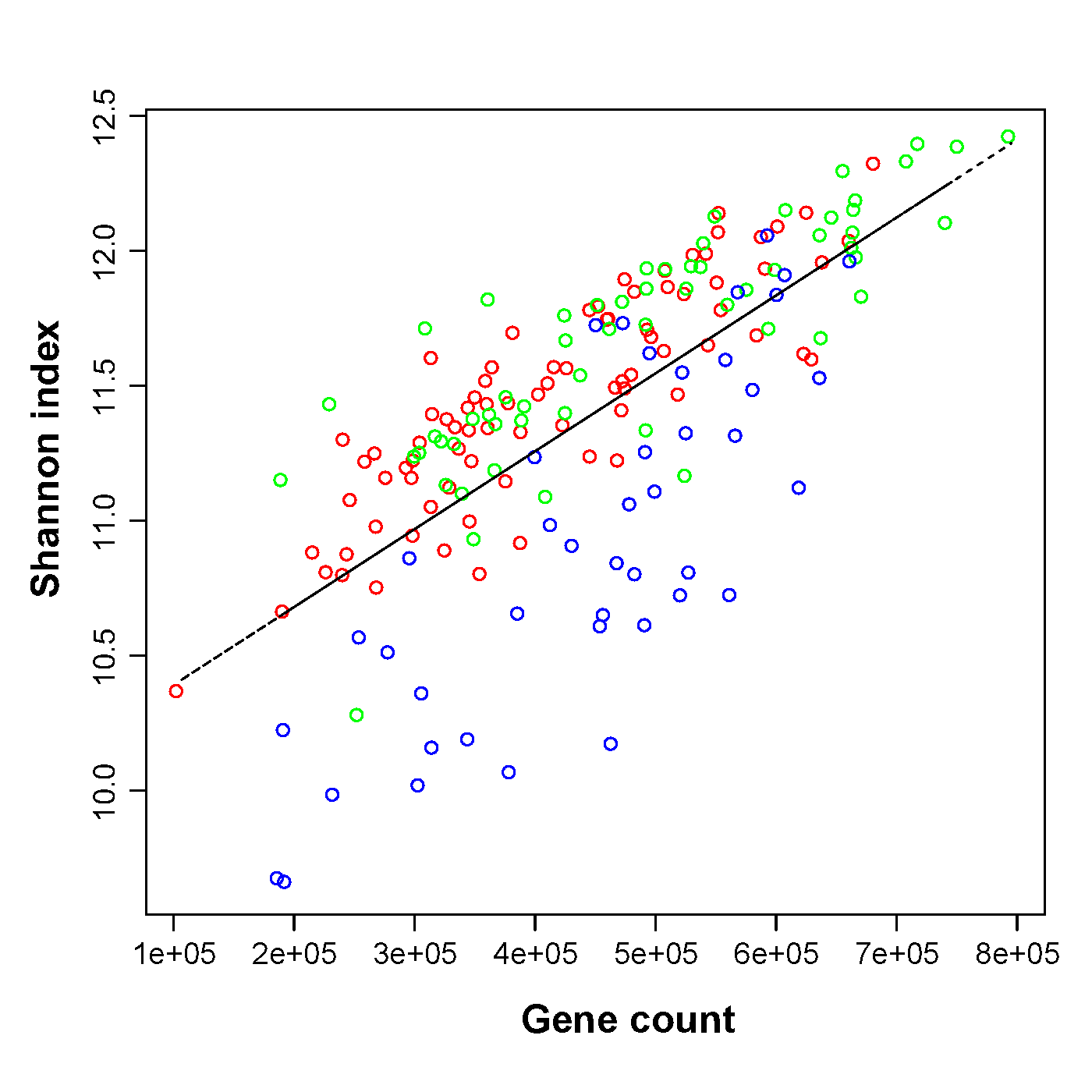


**Supplementary Figure 2.** Positive correlation between the gene count and Shannon index. Red circles: enterotype 1; Green circles: enterotype 2; Blue circles: enterotype 3.

| **a (beta diversity)** | **b (Hellinger distance)** |
| --- | --- |
| 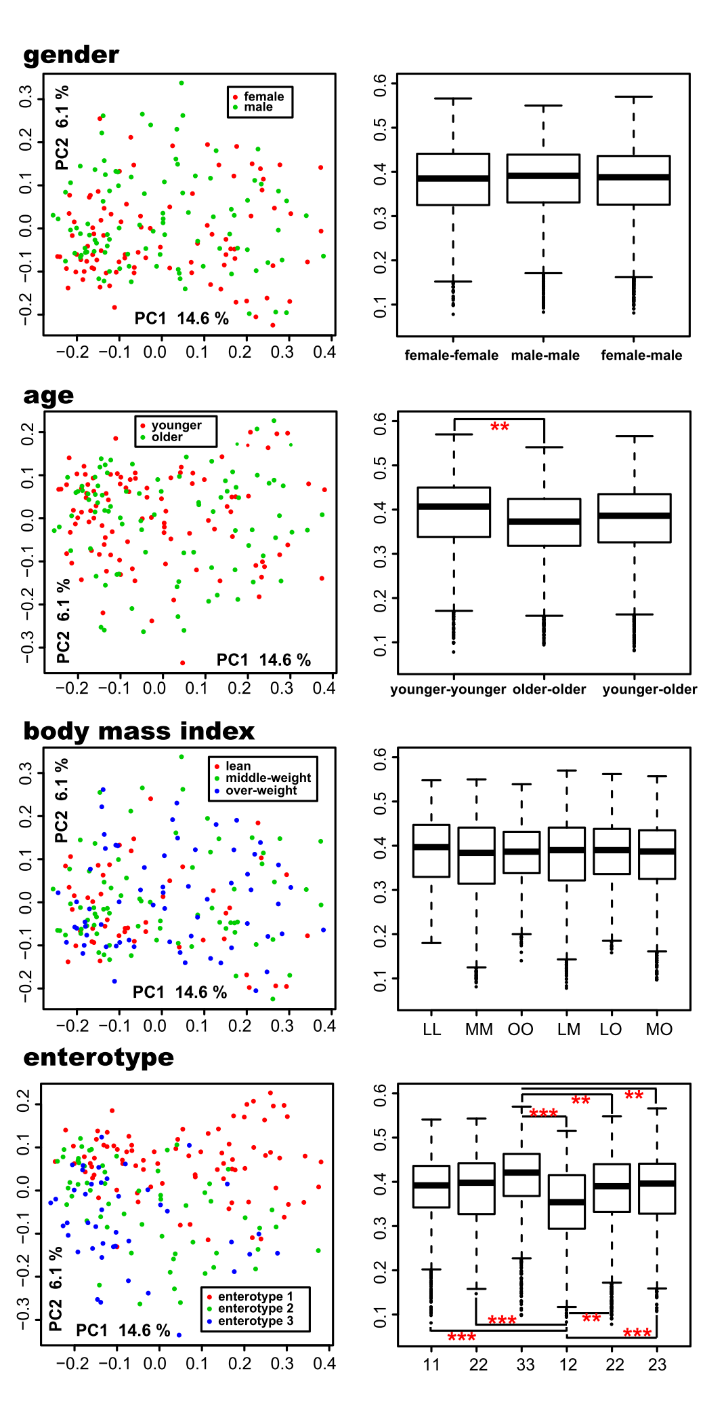 | 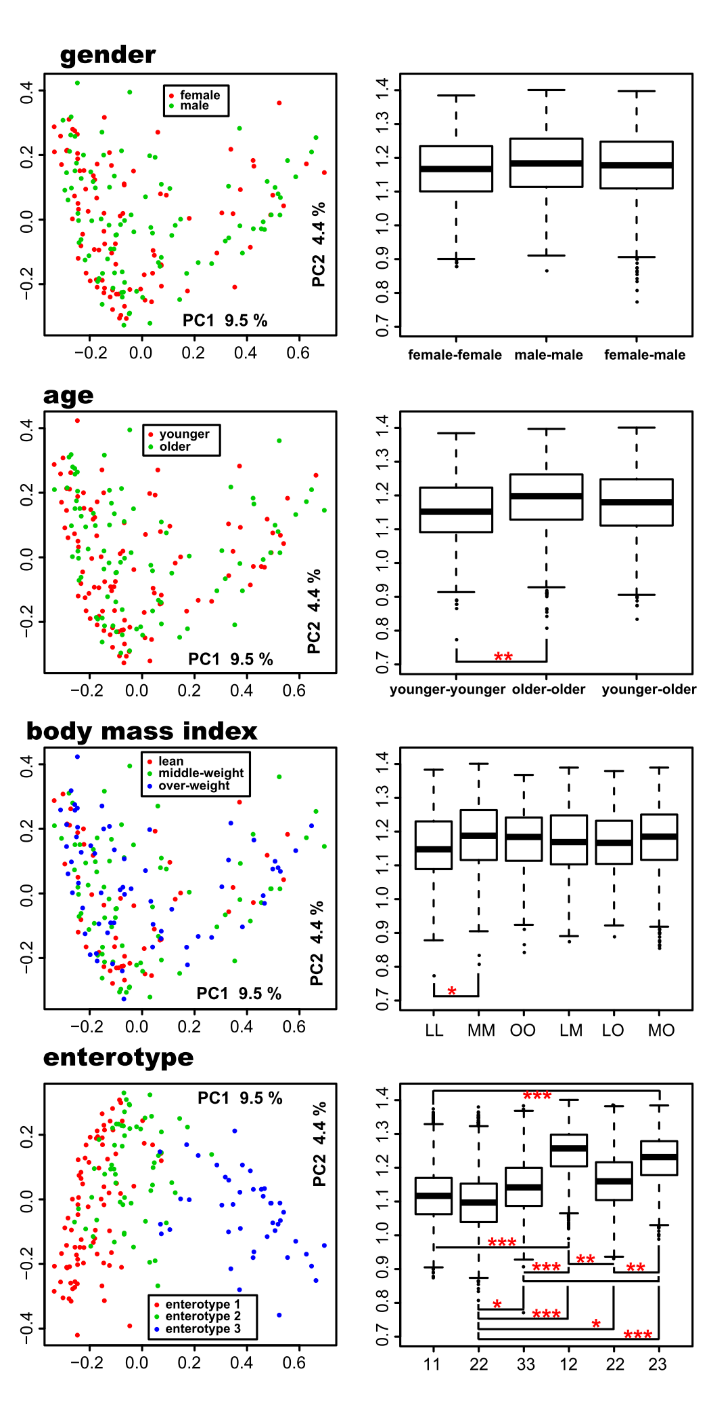 |

**Supplementary Figure 3.** Between-sample diversity analysis of 185 Chinese samples based on beta diversity (**a**) and Hellinger distance (**b**). The Principal Coordinate Analysis (PCoA) and average distance were showed in subgraphs. Student’s t-test: **P*<0.05; ***P*<0.01; ****P*<0.001.


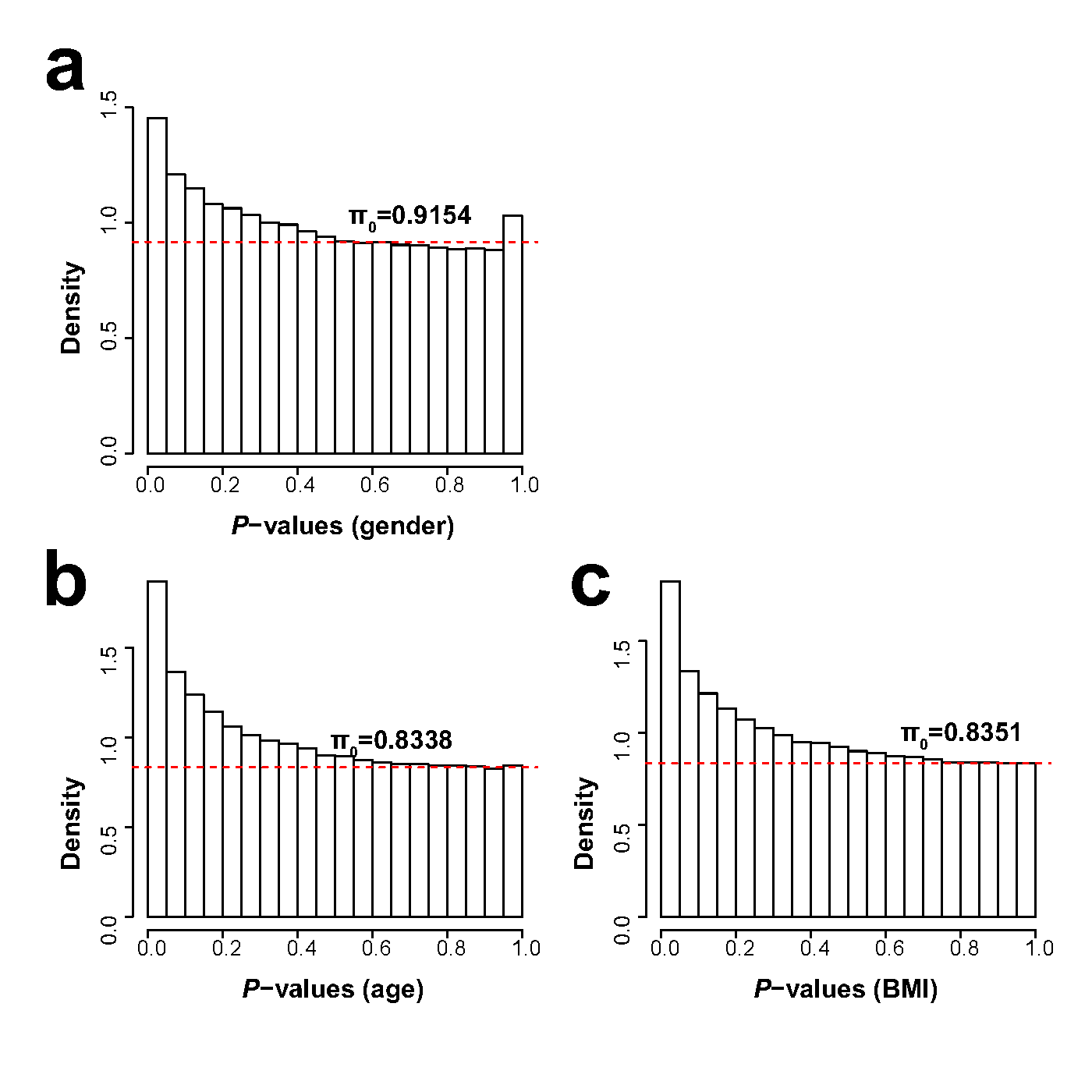


**Supplementary Figure 4.** *P*-values distribution of genes of each phenotype. (**a**): gender; (**b**): age; (**c**) body mass index. π_0_: the proportion of all tests in which the null hypothesis is true.


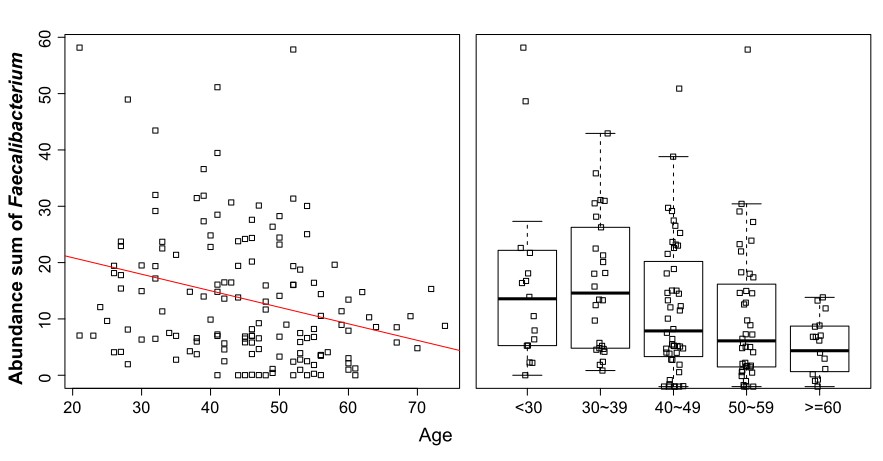


**Supplementary Figure 5.** Relative abundance of *Faecalibacterium* in 146 samples.

# Supplementary Tables

**Supplementary Table 1.** Phenotype information of 185 normal Chinese adults included in this study, grouped by enterotypes.

|  | **Female/Male** | **Age (mean±SD)** | **BMI (mean±SD)** |
| --- | --- | --- | --- |
| **Enterotype 1** | 44/39 | 43.7±13.3 | 23.1±3.3 |
| **Enterotype 2** | 17/26 | 40.7±12.3 | 23.3±3.6 |
| **Enterotype 3** | 29/30 | 41.5±12.5 | 22.8±3.4 |
| ***P* values** | 0.3579  (Fisher's exact test) | 0.2292  (Kruskal-Wallis test) | 0.8406  (Kruskal-Wallis test) |
|  |  |  |  |

**Supplementary Table 2.** Stratified sampling on the 185 samples for gender, age and BMI.

| **Groups** | **# samples** | **BMI [18,28]** | **AGE [20,60]** |
| --- | --- | --- | --- |
| male | 74 | 23.06 | 40.7 |
| female | 72 | 22.96 | 43.5 |
|  | | *P*=0.8252 (Student's test) | *P*=0.1363 (Student's test) |

**Gender:** we extracted 146 samples (74 male and 72 female, BMI≥18 and ≤28, age≥20 and ≤60) for gender, with no significant different in both BMI and age between males and females.

| **Groups** | **Mean±SD** | **# samples** | **AGE [20,60]** | **M/F** |
| --- | --- | --- | --- | --- |
| <20 (lean) | 18.51±1.04 | 33 | 36.1 | 15/18 |
| 20~25 (middle-weight) | 22.92±1.62 | 33 | 41.5 | 15/18 |
| >25 (over-weight) | 26.70±1.43 | 48 | 39.4 | 26/22 |
| *P*=3.0318e-23 (Kruskal-Wallis test) | | | *P*=0.05356  (Kruskal-Wallis test) | *P*=0.6646  (Fisher's exact test) |

**BMI:** We extracted 114 samples (33 lean, 33 middle-weight and 48 over-weight, age≥20 and ≤60) for BMI, with no significant different in both age and gender among the three groups.

| **Groups** | **Mean±SD** | **# samples** | **BMI [18,28]** | **M/F** |
| --- | --- | --- | --- | --- |
| <30 | 25.7±2.3 | 16 | 23.20 | 8/8 |
| 30~39 | 34.7±3.1 | 26 | 23.51 | 12/14 |
| 40~49 | 44.4±2.8 | 48 | 23.53 | 20/28 |
| 50~59 | 53.8±2.5 | 40 | 23.91 | 21/19 |
| >=60 | 64.4±4.8 | 16 | 22.95 | 9/7 |
| *P*=2.2018e-28 (Kruskal-Wallis test) | | | *P*=0.4935  (Kruskal-Wallis test) | *P*=0.8164  (Fisher's exact test) |

**Age:** We extracted 146 samples (5 groups, BMI≥18 and ≤28) of age, with no significant different in both BMI and gender among the five groups.

**Supplementary Table 3.** Number of identified genes for each phenotype.

|  | **No. of controlled samples** | **No. of genes (estimated FDR)** | | |
| --- | --- | --- | --- | --- |
|  |  | **Total (≥6 samples)** | ***P*≤0.05** | ***P*≤0.01** |
| **Age** | 146 | 1,904,252 | 178,024 (44.6%) | 51,596 (30.8%) |
| **BMI** | 114 | 1,795,100 | 163,788 (45.8%) | 45,041 (33.3%) |
| **Gender** | 146 | 1,898,669 | 137,998 (63.0%) | 35,850 (48.5%) |

**Supplementary Table 4.** Number of identified KOs for each phenotype.

|  | **No. of controlled samples** | **No. of genes (estimated FDR)** | | |
| --- | --- | --- | --- | --- |
|  |  | **Total (≥6 samples)** | ***P*≤0.05** | ***P*≤0.01** |
| **Age** | 146 | 5,578 | 631 (28.9%) | 177 (20.1%) |
| **BMI** | 114 | 5,558 | 251 (100%) | 48 (100%) |
| **Gender** | 146 | 5,583 | 439 (52.4%) | 119 (38.9%) |
